# Live high, train smart: translating altitude physiology to best practice with mechanistic insights

**DOI:** 10.3389/fphys.2026.1834288

**Published:** 2026-05-11

**Authors:** Raphaël Faiss, Olivier Girard

**Affiliations:** 1 Institute of Sports Sciences, University of Lausanne, Lausanne, Switzerland; 2 School of Human Sciences, University of Western Australia, Perth, WA, Australia

**Keywords:** altitude, athletes, hypoxia, performance, training

## Abstract

The first symposium on altitude training took place 60 years ago in Magglingen (Switzerland), where preparation for the 1968 Mexico Olympic Games provided “an excellent opportunity for the study of human adaptability” in hypoxic environments. Decades later, altitude or hypoxic training blocks -discrete periods (typically days to weeks) during which athletes reside, train, or intermittently expose themselves to hypoxia to stimulate physiological adaptations-have become a common component of elite endurance and team-sport training. Traditional strategies such as “live high-train high” and “live high-train low” have largely been adopted to stimulate erythropoiesis. The principal performance target is an increase in total hemoglobin mass (tHbmass), thereby enhancing O2 delivery to the working muscles through a higher arterial oxygen content. Despite ongoing debate regarding the precise physiological mechanisms underpinning altitude training adaptations, many key lessons were learned in the past 25 years related to hypoxic dose, exposure timing, and inter-individual variability in adaptative responses. Reflecting sustained scientific interest, no less than 12 comprehensive reviews or meta-analyses on altitude training strategies have been published in the past five years alone. These studies consistently report erythropoietic adaptations to hypoxia while seeking to refine practical recommendations regarding the optimal altitude severity and duration of exposure to potentiate increases in tHbmass. In this perspective article, potential vectors of performance improvement associated with hypoxic/altitude training were reconceptualized. By mapping contemporary altitude training strategies alongside their respective physiological mechanisms, we propose a framework that may help guide evidence-informed practice for research end-users seeking to boost their performance.

## Introduction: perspectives

The first symposium on altitude training took place 60 years ago in Magglingen (Switzerland), where preparation for the 1968 Mexico Olympic Games provided “an excellent opportunity for the study of human adaptability” in hypoxic environments (Weihe, 1965). Decades later, altitude or hypoxic training blocks -discrete periods (typically days to weeks) during which athletes reside, train, or intermittently expose themselves to hypoxia to stimulate physiological adaptations- have become a common component of elite endurance and team-sport training. Traditional strategies such as “live high-train high” (LHTH) and “live high-train low” (LHTL) have largely been adopted to stimulate erythropoiesis (Sharma, 2022; Bonato et al., 2023; Girard et al., 2023). The principal performance target is an increase in total hemoglobin mass (tHbmass), thereby enhancing O_2_ delivery to the working muscles through a higher arterial oxygen content (Lundby and Robach, 2025). Despite ongoing debate regarding the precise physiological mechanisms underpinning altitude training adaptations, many key lessons were learned in the past 25 years related to hypoxic dose, exposure timing, and inter-individual variability in adaptative responses (Girard et al., 2023). Reflecting sustained scientific interest, no less than 12 comprehensive reviews or meta-analyses on altitude training strategies have been published in the past five years alone (Seitz et al., 2020; Benavente et al., 2023; Bonato et al., 2023; Chen et al., 2023; Feng et al., 2023; Faiss et al., 2024; Feng et al., 2024; Ramchandani et al., 2024; Boulares et al., 2025; Deng et al., 2025; Lundby and Robach, 2025; Chen et al., 2026). These studies consistently report erythropoietic adaptations to hypoxia while seeking to refine practical recommendations regarding the optimal altitude severity and duration of exposure to potentiate increases in tHbmass.

In this perspective article, we aim to reconceptualize the potential vectors of performance improvement associated with hypoxic/altitude training. By mapping contemporary altitude training strategies alongside their respective physiological mechanisms, we propose a framework that may help guide evidence-informed practice for research end-users seeking to boost their performance.

## Idiosyncratic responses and potential negative mechanisms of altitude training

A striking degree of interindividual variability in physiological responses to altitude exposure has consistently been revealed over the past 60 years of research on altitude training. Substantial heterogeneity in tHbmass and performance-related adaptations has been reported across different altitude strategies, exposure durations, and athlete populations. Notably, only around half of the altitude training studies report increased performance or favorable physiological adaptations, while negative mechanisms (e.g., fatigue, decrease in muscle mass or immunodepression) were observed more often with prolonged hypoxic exposures (**Table 1**).

**Table 1:** Overall efficacy of various hypoxic training methods [data derived from (Seitz et al., 2020; Sharma, 2022; Benavente et al., 2023; Bonato et al., 2023; Feng et al., 2023; Faiss et al., 2024; Feng et al., 2024) to update original table presented in (Millet et al., 2010)].

Hypoxic training method	Number of studies	Increased performance/favorable mechanisms	No additional effect	Negative mechanisms
LHTH	17	6 35%	7 41%	4 24%
LHTL	42	23 55%	8 19%	11 26%
Continuous hypoxic training (CHT)	18	4 22%	13 72%	1 6%
Intermittent hypoxic training (IHT)	20	10 50%	10 50%	0
Resistance training in hypoxia (RTH)	17	3 18%	11 64%	3 18%
Repeated Sprint Training in Hypoxia (RSH)	22	18 82%	4 18%	0
All	136	47%	42%	14%

A detailed table with all studies is available as **Supplementary File**.

The implicit assumption that the effectiveness of altitude training can be predicted primarily from the exposure method or hypoxic dose alone is thereby challenged. In practice, two athletes exposed to the same hypoxic dose may experience markedly different physiological stimuli with adaptive responses. The prevailing focus on training modality and dose-response relationships may hence overlook a more fundamental issue: a complex network of idiosyncratic physiological mechanisms mediates adaptations to hypoxia. Factors such as iron availability, hypoxia-inducible factor signaling, erythropoietin sensitivity, ventilatory acclimatization, sleep quality, training load tolerance, and individual genetic predisposition may all influence the extent to which altitude exposure translates into meaningful functional adaptations and ultimately performance improvements (Sharma, 2022).

The addition of hypoxic stimuli to further promote physiological responses during “live low train high” altitude training modalities (e.g., after CHT, IHT, RTH or RSH, see **Table 1**) support efforts to include these methods within training regimen, as they appear to carry limited risk of negative mechanisms, despite no transcendent performance gains (Faiss et al., 2024). On the other hand, prolonged hypoxic exposures (e.g., LHTH or LHTL) remain widely used despite contentious evidence regarding their systematic effectiveness for a given hypoxic dose, largely due to significant inter-individual variability in responses.

Ultimately, the central objective remains improving athletic performance. Hypoxic training strategies may target either quantitative adaptations (i.e., increasing the oxygen-carrying capacity of the blood via higher tHbmass) or qualitative adaptations (i.e., improving oxygen diffusion and use at the muscular level) (Lundby et al., 2017). Historically, hematological adaptations have been considered the primary mediator of performance enhancement after altitude training (Levine and Stray-Gundersen, 2005). An important conceptual advance was the proposal to quantify a “hypoxic dose” (km.h^-1^) calculated from the time spent (hours) at a given altitude (km), allowing to estimation of the expected tHBmass gain (Garvican-Lewis et al., 2016). Importantly, this metric also provides a standardized framework for comparing altitude-training studies that differ in exposure severity and duration, enabling a more objective interpretation of hypoxic interventions across experimental and applied settings.

## Limitations of the “hypoxic dose” paradigm

This perspective argues that the prevailing paradigm, centered on optimizing and quantifying hypoxic dose, may be insufficient to fully explain or predict the benefits of chronic altitude exposure. Practically, plotting tHbmass gains as a function of time spent at altitude has helped generate applied guidelines regarding the duration and elevation required to elicit measurable hematological adaptations, particularly changes must exceed the measurement error (nowadays below 2%) of the carbon monoxide rebreathing method used to quantify tHbmass (Chapman et al., 2014). However, the assumption of a linear relationship between hypoxic dose and tHbmass gain is likely unrealistic. First, the erythropoietin response to moderate altitude is finite, with tHbmass increases eventually reaching a plateau after several weeks of acclimatization. Second, hypoxic dose cannot be universally applied across athletes irrespective of their training background and altitude history, simply because it does not reflect the individual physiological stress imposed by a given hypoxic stimulus (Millet et al., 2016). For instance, altitude training camps conducted above 2000 m for ~3 weeks (i.e., representing a common hypoxic dose of approximately 1000 km.h^-1^) have been associated with tHbmass ranging between 0 and + 6%, illustrating the large inter-individual variability and the need to individualize altitude training beyond hypoxic dose alone.

**Figure 1:**
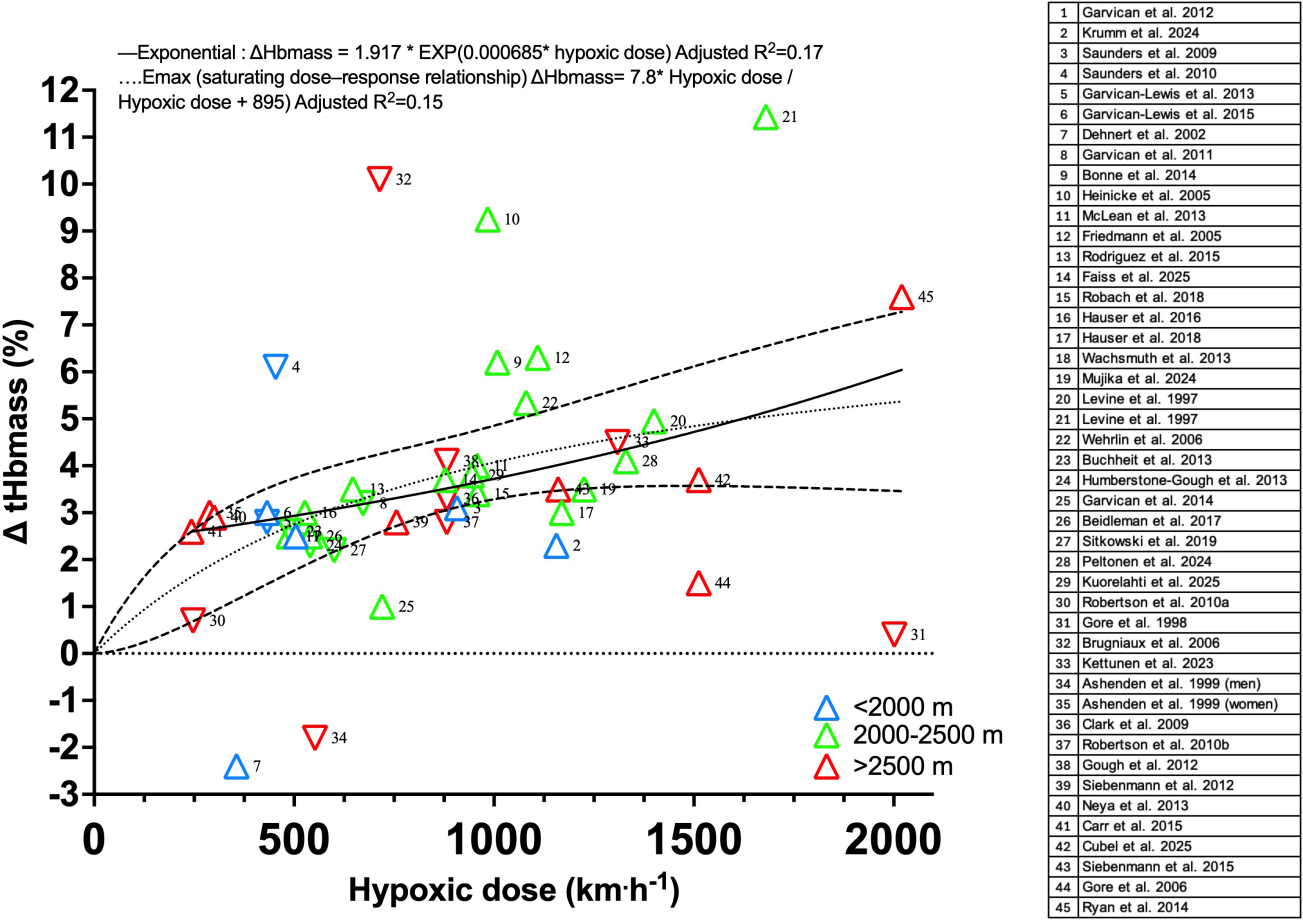
Changes in total hemoglobin mass (tHbmass) as a function of accumulated hypoxic dose [as defined in (Garvican-Lewis et al., 2016)] reported for altitude training studies since 1997. Triangle colors indicate the altitude level of exposure. Upright and inverted triangles correspond to exposures to hypobaric hypoxia or normobaric hypoxia, respectively. The corresponding studies were retrieved from indexed publications cross-referenced in (Bonato et al., 2023; Chen et al., 2023; Feng et al., 2023; Ramchandani et al., 2024; Boulares et al., 2025; Deng et al., 2025; Lundby and Robach, 2025; Chen et al., 2026). Concentration-response data were analyzed by non-linear regression using GraphPad Prism 11 (GraphPad Software, Boston, MA, USA). Saturating dose-response relationships were fitted to a hyperbolic Emax model (dotted line with 95% confidence intervals (dashed line): E = (Emax × D)/(EC50 + D), where E is the measured effect at Dose D, Emax is the maximum asymptotic effect, and EC50 is the hypoxic dose producing half-maximal effect. Time-dependent growth data were fitted to an exponential growth model (solid line): Y = Y_0_ × e^(k×D), where Y_0_ is the initial value at the lowest dose reported, k is the rate constant, and D the hypoxic dose. Variation of residuals was assumed Gaussian with a similar standard deviation along the increase in hypoxic dose. Both models were fitted by least-squares minimization, and goodness of fit was assessed by the coefficient of determination (R²).

To reassess the hypoxic dose-tHbmass relationship using studies published over the past decade, altitudes between 2000 and 2500 m appear to provide a practical compromise for altitude camps (**Figure 1**). The available evidence suggests a saturating dose–response relationship, whereby tHbmass increases with hypoxic dose but approaches an asymptotic maximum (i.e. consistent with an Emax-type model), rather than a simple linear relationship.

Indeed, hypoxic dose appear to explain only a small proportion of the variance in Hbmass responses (R² = 0.17), reinforcing the well-documented inter-individual variability in altitude adaptive responses (Lundby and Robach, 2025). Furthermore, observations of athletes who fail to improve performance despite large increases in tHbmass [after either altitude training (Cubel et al., 2025) or erythropoietin treatment (Bonne et al., 2025)] illustrate that hematological adaptations alone may not represent the ultimate target for performance gains with altitude training.

## Towards a mechanistic and individualized framework for altitude training

Eventually, only performance matters. The wide range of contemporary altitude training strategies (Girard et al., 2017; Girard et al., 2020) reflects the tremendous development of hypoxic training methods over the past decades. Yet, a clear translational framework is still lacking to identify which physiological mechanisms underpin the performance benefits associated with each hypoxic method. Moving beyond the recurring observation that athletes respond differently – or sometimes not at all – to various hypoxic training strategies is paramount. Although many of the mechanisms involved in the response to hypoxic training have been identified, their practical implementation requires careful planning and consideration of multiple interacting factors.

**Figure 2:**
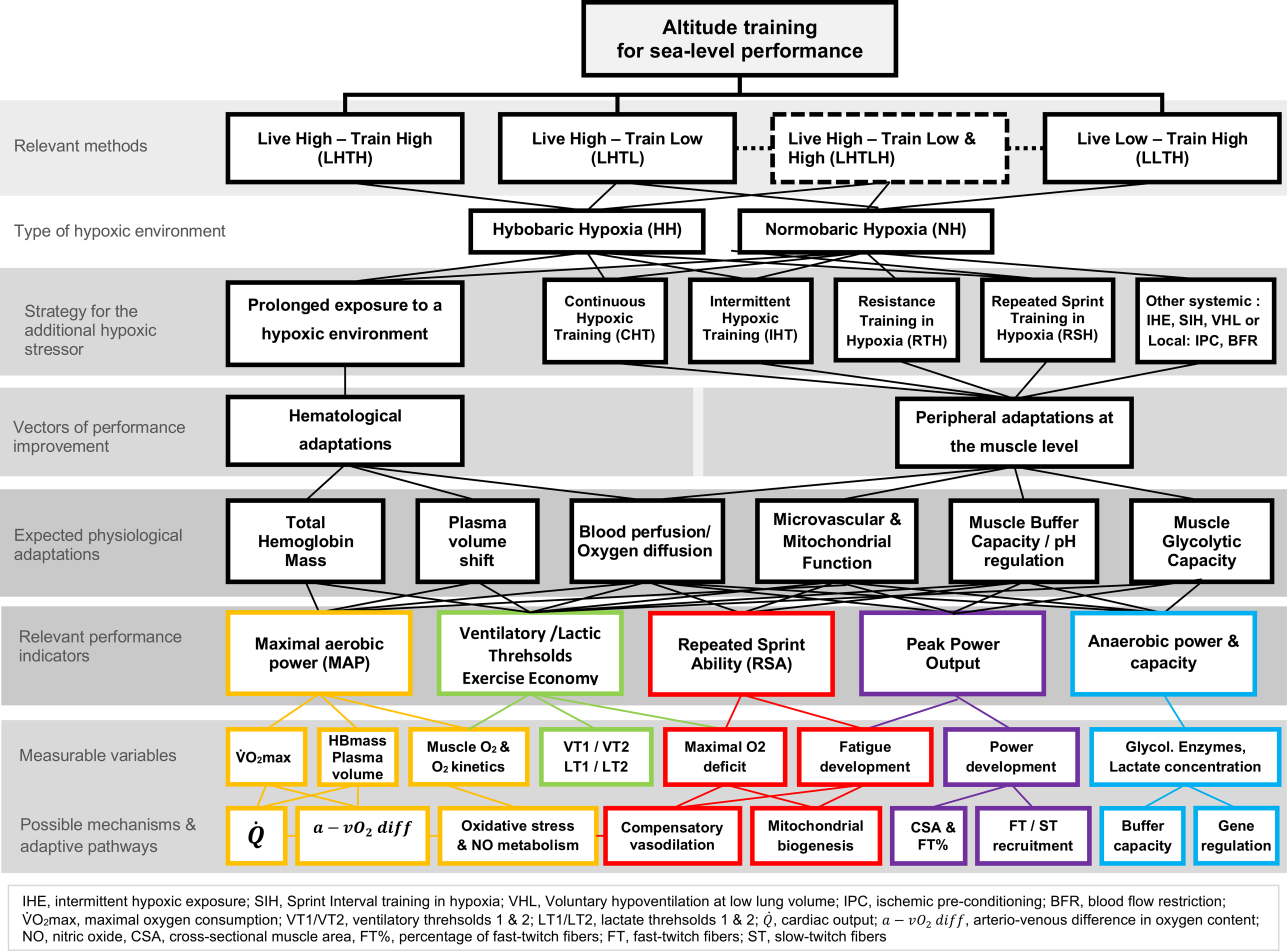
Mechanistic panorama of altitude training methods and their potential vectors of performance improvement. The framework links hypoxic training methods with underlying physiological adaptations which could influence performance. These purported physiological adaptations may translate into key performance indicators for coaches and athletes, assessed with commonly measured variables. The panorama may also help identify individual adaptive pathways that could be targeted by a given hypoxic intervention. The color scheme is for illustrative purposes only, and the connections between boxes reflect documented or hypothesized associations based on current scientific evidence.

We introduce a mechanistic framework in which responses to altitude training are viewed as inherently idiosyncratic and biologically mediated, rather than determined solely by exposure metrics such as hypoxic dose (**Figure 2**).

Our mechanistic panorama first identifies variables reported in previous studies on the effects of altitude/hypoxic training in humans. Possible mechanisms and adaptive pathways were then added where they have been previously identified or investigated in exercise training studies (with or without hypoxia). This approach enabled the identification of relevant performance indicators related to these physiological adaptations. The selection of an adequate hypoxic training strategy may ultimately be guided by aligning the chosen method with the targeted performance outcome. This framework was conceptualized from the individual variables reported in several comprehensive reviews and meta-analyses published recently (Seitz et al., 2020; Benavente et al., 2023; Bonato et al., 2023; Chen et al., 2023; Feng et al., 2023; Faiss et al., 2024; Feng et al., 2024; Ramchandani et al., 2024; Boulares et al., 2025; Deng et al., 2025; Lundby and Robach, 2025; Chen et al., 2026). By shifting the emphasis towards the physiological mechanisms that mediate adaptations to hypoxia, researchers and practitioners may better understand why some athletes exhibit limited responses to chronic altitude exposure whereas others show robust responses, highlighting the need for more individualized training regimens.

A holistic approach is therefore necessary to integrate targeted physiological adaptations into the decision process surrounding the use of altitude training. Such a bottom-up approach also offers the opportunity to combine different strategies (e.g., heat or hypoxia) either concurrently, sequentially or simultaneously considering the magnitude of the individual response for a given outcome variable (Girard et al., 2024). Further research should aim to identify more robust markers of systemic performance adaptations beyond relying solely on unequivocal tHbmass gains after applying any so-called ‘Gold standard’ altitude strategies (i.e. LHTH or LHTL). Finally, because athletes often face practical trade-offs between training efficacy, available time, and travel and/or financial constraints – and because multiple training strategies cannot always be applied simultaneously – this perspective may help guide the practical selection of an adequate altitude training strategy and inform decision-making when selecting the most appropriate method.

## Data Availability

The raw data supporting the conclusions of this article will be made available by the authors, without undue reservation.
